# Machine Learning-Enhanced MALDI-TOF Mass Spectrometry for Screening HBsAg-Positive Patients

**DOI:** 10.3390/microorganisms14030702

**Published:** 2026-03-20

**Authors:** Tiantian Zhang, Shixuan Huang, Junxun Li, Yuwei Wu, Xinyu Zhao, He Gao, Juan Yang, Lingshuang Yang, Lulu Cao, Xinqiang Xie, Hui Zhao, Jing Cheng, Hongxia Tan, Ying Li, Qingping Wu

**Affiliations:** 1 School of Food and Biological Engineering, Shaanxi University of Science and Technology, Xi’an 710021, China; ttsghww@163.com; 2 State Key Laboratory of Applied Microbiology Southern China, Guangdong Provincial Key Laboratory of Microbial Safety and Health, National Health Commission Science and Technology Innovation Platform for Nutrition and Safety of Microbial Food, Key Laboratory of Big Data Technologies for Food Microbiological Safety, State Administration for Market Regulation, Institute of Microbiology, Guangdong Academy of Sciences, Guangzhou 510070, Chinajuanayangon@163.com (J.Y.); yangls8272@163.com (L.Y.);; 3 Food and Drug Laboratory, Guangdong Detection Center of Microbiology, Guangzhou 510070, China; 4 Department of Medical Laboratory, First Affiliated Hospital, Sun Yat-Sen University, Guangzhou 510080, China

**Keywords:** hepatitis B virus, hepatitis B surface antigen, matrix-assisted laser desorption/ionization time-of-flight, serum, machine learning

## Abstract

Hepatitis B virus (HBV) remains a major global public health challenge, and its early screening is essential for controlling transmission and improving treatment outcomes. We analyzed serum samples from 422 participants via Matrix-assisted laser desorption/ionization time-of-flight mass spectrometry (MALDI-TOF MS) to establish a screening model for hepatitis B surface antigen (HBsAg)-positive status. Following multi-bin preprocessing and single-sample spectral aggregation, we assessed three machine learning algorithms—random forest, deep neural network, and light gradient boosting machine (LightGBM). Among them, the LightGBM model achieved the best performance, with an optimized F1 score of 0.87 and an area under the receiver operating characteristic curve (AUC) of 0.94. A 100-iteration ensemble feature stabilization strategy identified twelve distinct *m*/*z* peaks as stable biomarkers for HBsAg-positive screening. Independent validation yielded sensitivity of 77.7% and specificity of 76.0%—insufficient for individual diagnosis but potentially suitable for population-level surveillance programs combined with confirmatory testing, particularly in resource-limited settings where conventional methods are impractical. Notably, the method offers a detection time of approximately one minute, a per-sample cost of ~$0.14. In conclusion, the combination of MALDI-TOF MS and machine learning enables a rapid, low-cost screening tool for large-scale HBV detection.

## 1. Introduction

Hepatitis B virus (HBV) infection is one of the most serious public health problems worldwide [1]. According to the World Health Organization (WHO), an estimated 300,000,000 individuals worldwide are chronically infected with HBV, with approximately 820,000 deaths annually attributed to HBV-related complications, including cirrhosis and hepatocellular carcinoma [2,3]. Although vaccination has achieved remarkable results in reducing new infections, the number of patients with latent and chronic HBV infection is still large in many high-endemic areas, and there are often no obvious clinical symptoms in the early stages of infection, resulting in frequent missed diagnosis and delayed treatment. Therefore, developing detection technologies that can identify HBV infection early, quickly, and accurately is crucial for reducing the threat of viral hepatitis to public health.

Currently, clinical HBV testing relies primarily on serological markers and molecular biological methods. The enzyme-linked immunosorbent assay (ELISA) remains one of the most commonly used platforms for HBV serological diagnosis, alongside other widely applied techniques such as chemiluminescence immunoassay and immunochromatographic assays [4]. These methods assess infection status by detecting hepatitis B virus surface antigen (HBsAg), hepatitis B virus e antigen (HBeAg), and related antibodies [5]. They offer the advantages of simplicity and low cost. However, their sensitivity may be reduced in patients with viral mutants, low viral loads, and those in the immune-tolerant phase, leading to false-negative results [6]. Nucleic acid testing methods, such as real-time quantitative polymerase chain reaction (qPCR), are considered the gold standard for clinical viral diagnostics owing to their high analytical sensitivity and accuracy [7], and the global COVID-19 pandemic has further promoted their widespread implementation, positioning qPCR as a core technical platform for emergency large-scale infectious disease screening worldwide [8]. However, this method relies on sophisticated temperature control equipment and has a relatively long detection cycle, which limits its widespread application in clinical and public health fields, especially in resource-constrained areas [9].

In recent years, mass spectrometry-based detection technologies have provided new insights into clinical pathogen detection and disease diagnosis. Matrix-assisted laser desorption/ionization time-of-flight mass spectrometry (MALDI-TOF MS) has been widely used in clinical microbial identification due to its advantages, including rapid analysis, high throughput, minimal sample requirements, and the absence of complex sample pretreatment [10]. MALDI-TOF MS can quickly capture biomarker fingerprints of numerous characteristic molecules within minutes [11]. These fingerprints encompass known pathogen-associated molecules as well as unknown features of the target. Consequently, applying MALDI-TOF MS to serological testing for HBV infection holds promise for overcoming the limitations of traditional methods and enabling rapid, noninvasive, and multidimensional analysis of infection status [12,13,14,15]. However, most previous MALDI-TOF MS studies for HBV detection have relied on manual analysis or conventional statistical methods such as principal component analysis and partial least squares discriminant analysis [16]. These approaches are ill-equipped to handle large-scale, high-dimensional, and high-noise spectral datasets, resulting in models with poor generalizability and uninterpretable diagnostic outputs, failing to discern differential features from complex background signals, which ultimately limits diagnostic performance [10].

The development of machine learning (ML) provides a powerful tool for addressing this challenge. ML can automatically extract underlying patterns from large-scale, high-dimensional data and construct classification models with strong generalization capabilities [17]. Algorithms such as gradient boosting (e.g., light gradient boosting machine (LightGBM)) and deep neural networks (DNNs) have demonstrated excellent classification and prediction performance in genomics, metabolomics, and clinical imaging analysis [15,16,18]. Compared to traditional statistical analysis, ML algorithms are superior in processing nonlinear relationships and high-noise data. In MALDI-TOF MS data analysis, ML can efficiently identify characteristic peaks that are highly correlated with disease states and improve diagnostic sensitivity and specificity by continuously optimizing feature weights. However, clinical datasets in infectious disease screening are frequently constrained by limited sample sizes, which poses a significant challenge for model training and generalization. To address this, three algorithms with complementary mechanisms for handling high-dimensional, small-sample data were selected in this study. LightGBM is well-suited for such settings because its built-in regularisation parameters directly constrain tree complexity, reducing the risk of overfitting when training samples are limited. RF tolerates small sample sizes through bootstrap aggregation and random feature subsampling, which reduce model variance by constructing multiple decorrelated trees from different subsets of samples and features. DNN is included to capture cross-channel composite features from the physically continuous signals of MALDI-TOF MS, where the aggregation of 24 technical replicate spectra per sample prior to model input substantially increases the information content of each training instance and early stopping is applied to control overfitting given the limited training set.

Building on these algorithmic foundations, this study combines MALDI-TOF MS with machine learning (ML-MALDI-TOF MS) to develop a rapid, low-cost screening model for large-scale detection of HBsAg-positive HBV infection. The model integrates single-sample spectral aggregation and standardization, multi-bin preprocessing (3, 5, 10, and 15 *m*/*z*), optimization of the minimum number of spectral replicates per sample, and a 100-iteration SHAP-based feature stabilization strategy. This approach addresses limitations of conventional spectral analysis, enabling efficient processing of large-scale datasets and robust identification of stable screening features. The resulting framework provides a reproducible paradigm for integrating mass spectrometry with interpretable machine learning in infectious disease diagnostics and offers potential utility for population-level HBV screening and early detection in public health applications.

## 2. Materials and Methods

**Figure 1:**
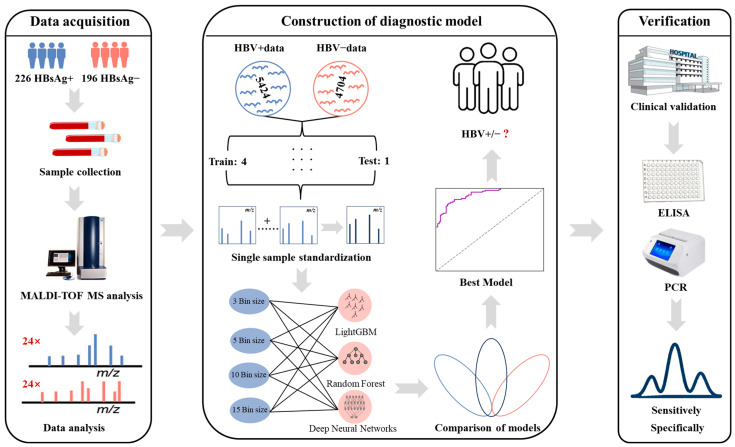
MALDI-TOF MS-based HBsAg-positive HBV infection screening workflow. Serum samples from 422 subjects were analyzed using MALDI-TOF MS. Following data standardization, multiple machine learning algorithms were employed to train and optimize screening models. The best-performing model was selected and subsequently validated on an independent clinical cohort.

This study constructed a system for identifying HBsAg-positive patients based on MALDI-TOF MS combined with ML. The detailed workflow is shown in Figure 1. Initially, serum samples from 422 participants yielded 10,128 protein spectra (5424 originating from 226 HBsAg-positive individuals and 4704 from 196 HBsAg-negative controls) to establish the reference database. Three different ML algorithms were applied to develop the primary models based on this dataset, along with 4 sub-datasets with different bin sizes of the fingerprint proteins. Subsequently, the best-performing model was selected and its hyperparameters were adjusted for improvement. Finally, the actual detection capability of the refined model was evaluated on an independent cohort of 20 volunteers.

### 2.1. Clinical Cohort

Serum samples were collected at the First Affiliated Hospital of Sun Yat-sen University from 1 November 2023 to 30 April 2024 for the construction of the screening model for HBsAg-positive HBV infection. Another 20 healthy volunteers were recruited from 1 July to 31 July 2024 for performance assessment of the model. The HBV infection status was confirmed using the HBsAg qualitative test kit (Abbott, Kilkenny, Ireland), and five core liver function parameters—alanine aminotransferase (ALT), aspartate aminotransferase (AST), albumin (ALB), direct bilirubin (DBIL), and total bilirubin (TBIL)—were evaluated using the AU5800 system (Beckman Coulter, Brea, CA, USA). Among these, 226 samples tested positive for HBsAg, all positive samples were obtained from untreated HBsAg-positive patients without cirrhosis, hepatocellular carcinoma, or other end-stage complications. To further assess the specificity of our model for other blood-borne infections, we included two HBsAg-negative control samples, one from a patient with syphilis (Treponema pallidum-positive) and one from a patient with hepatitis C virus (HCV). Additionally, HBV viral particles were concentrated from cell culture supernatants (HepG2.2.15, HepG2-NTCP infected with HBV genotype B or C), and purified HBV serological markers were analyzed to identify characteristic peaks detected by the model. This study was conducted according to the guidelines of the Declaration of Helsinki and was approved by Institutional Review Board of the first affiliated hospital, Sun Yat-sen University ([2023]463FASH20240342).

### 2.2. MALDI-TOF Spectra Acquisition

1 μL of serum was put onto a MALDI-TOF target plate (Bruker, Bremen, Germany) and air-dried at room temperature. Then 1 μL of 70% formic acid (Sigma-Aldrich, St. Louis, MO, USA) and 1 μL of α-cyano-4-hydroxycinnamic acid matrix solution (10 mg/mL, 50% acetonitrile/2.5% trifluoroacetic acid, Bruker) was added and air-dried on the plate sequentially. The target plate was placed into the MALDI-TOF mass spectrometer (Microflex Smart, Bruker) with Compass flexControl (Version 1.4, Bruker) for further analysis. Each sample was positioned eight times on the target plate. With each position measured for three times, the mass spectrometer was run in linear positive mode, collecting the spectrum from each spot at a laser frequency of 60 Hz, covering a molecular mass range of 2000 to 20,000 *m*/*z*.

### 2.3. Data Pre-Processing and Dataset Preparation

Raw mass spectrometry data were extracted and processed using R packages “MALDIquant” (version 1.18) and “MALDIquantForeign” (version 0.14.1). This workflow encompassed quality control, data calibration, smoothing, baseline reduction, normalization, and peak identification. Particularly, the intensity data were acquired between 2000 and 20,000 *m*/*z*, and the raw spectral data were then processed. To mitigate peak shift variability inherent to MALDI-TOF MS and enhance feature reproducibility, we generated four parallel feature sets using bin sizes of 3, 5, 10, and 15 *m*/*z* for downstream analysis and model comparison. This binning strategy aggregates ion signal intensities within fixed *m*/*z* intervals, thereby reducing the impact of *m*/*z* peak shifts caused by instrumental voltage fluctuations, laser energy variations, and matrix effects. By consolidating signals from the same molecular feature that might otherwise be fragmented across adjacent *m*/*z* channels due to minor shifts, this approach improves the stability and reproducibility of spectral data for subsequent machine learning analysis.

All 24 technical replicate spectra from each serum sample were aggregated to generate representative proteomic profiles and account for technical variability inherent in MALDI-TOF MS. This single-sample standardization reduces random noise, improves the signal-to-noise ratio, and stabilizes downstream spectral features. To prevent data leakage and ensure unbiased model evaluation, dataset partitioning was performed at the patient level. The 422 participants were first stratified by HBsAg status, then randomly assigned to training (60%) or test (40%) sets within each stratum. All 24 aggregated spectra from a given patient were assigned exclusively to the corresponding dataset. After data standardization, t-distributed stochastic neighbor embedding (t-SNE) was applied using scikit-learn (Python 3.8) with a perplexity parameter of 30 for dimensionality reduction and visualization of spectral patterns.

### 2.4. Model Training, Interpretability Analysis and Model Optimization

The preliminary prediction models were built based on three different algorithms, RF, DNN, and LightGBM, using the keras library and the lightgbm library. These models process input spectra to generate a *predict* value for HBsAg-positive status. *Predict* is a crucial statistic for determining both the minimum spectral replicates required per sample and the confidence of infection classification. To establish the optimal number of technical replicates for reliable prediction, we conducted a systematic optimization experiment. Six serum samples were selected at random, including three HBsAg-positive and three HBsAg-negative individuals. For each sample, spectral replicates were incrementally increased from 1 to 24, and the *predict* value was recorded at each step. Progressive improvement in the *predict* value was observed with additional replicates, reflecting noise reduction through signal averaging. The optimal replicate number was determined at the stabilization point where further increases yielded no significant benefit, achieving an optimal balance between spectral reliability and acquisition efficiency. This standard was subsequently adopted for all sample collections, ensuring sufficient spectral information for robust prediction while minimizing unnecessary data acquisition.

In model training, early stopping was applied as the callback strategy, and the difference between the actual labels and the model’s predictions is measured using logloss. The training process was terminated after 100 consecutive epochs if the model does not exhibit improvement in binary logloss. Additionally, the two primary measures used to evaluate the model’s performance were the area under the curve (AUC) and the F1 score. These metrics offer a thorough evaluation of the accuracy of the model from multiple perspectives.

Using the SHAP (Shapley additive explanation) library, we established a framework for interpretable feature analysis [19]. SHAP quantifies each feature’s marginal contribution to model predictions, providing insights into decision-making. However, values from a single model may be sensitive to stochastic elements such as random seed initialization and data shuffling, leading to unstable feature rankings. To address this, we constructed 100 independent models with identical training data and hyperparameters but distinct random seeds. SHAP analysis was performed for each model, and only features with consistently non-zero contributions across all iterations were retained. This consensus-based filtering reduces noise-driven variability, minimizes sensitivity to random initialization, and ensures that selected features represent stable, biologically relevant spectral signals, resulting in a reliable and reproducible screening model for HBsAg-positive HBV infection.

### 2.5. Diagnostic Procedures of the Optimized Model

The fingerprints of a single serum sample is fed into the final screening model for HBsAg-positive HBV infection, which then calculates the *predict* value. The model classifies the sample as positive for HBsAg-positive HBV infection when *predict* ≥ 0.5 (classified as Positive). On the other hand, *predict* < 0.5 indicates that the sample originates from an individual with HBsAg-negative HBV infection.

### 2.6. Statistical Analysis

Statistical analyses were performed using GraphPad Prism (version 9.5; Dotmatics, San Diego, CA, USA). Categorical data were analyzed using the Chi-square test, pairwise comparisons were conducted using McNemar’s test with continuity correction, and continuous non-parametric data were compared using the Mann–Whitney U test. *p* < 0.05 was considered statistically significant.

## 3. Results

### 3.1. Demographic Characteristics of the Volunteers

Baseline demographic and clinical characteristics of the study population are summarized in Table S1. No significant differences were observed between the HBsAg-positive (*n* = 226) and HBsAg-negative (*n* = 196) groups in terms of age (51.23 ± 14.12 vs. 50.14 ± 16.80 years, *p* = 0.40) or sex distribution (141/85 vs. 120/76 male/female, *p* = 0.81). Analysis of liver function parameters revealed no statistically significant differences between the two groups for ALB (*p* = 0.28), DBIL (*p* < 0.05), TBIL (*p* = 0.16), ALT (*p* = 0.07), or AST (*p* = 0.06). These results demonstrate that the HBsAg-positive and HBsAg-negative cohorts were well-matched in age, sex, and baseline liver function. The absence of significant differences in these key demographic and clinical variables indicates that subsequent model performance—distinguishing between the two groups—can be attributed to HBV-associated spectral signatures captured by MALDI-TOF MS rather than confounding factors related to age, sex, or underlying liver dysfunction.

### 3.2. Basic Information of the Serum Spectrum Database

**Figure 2:**
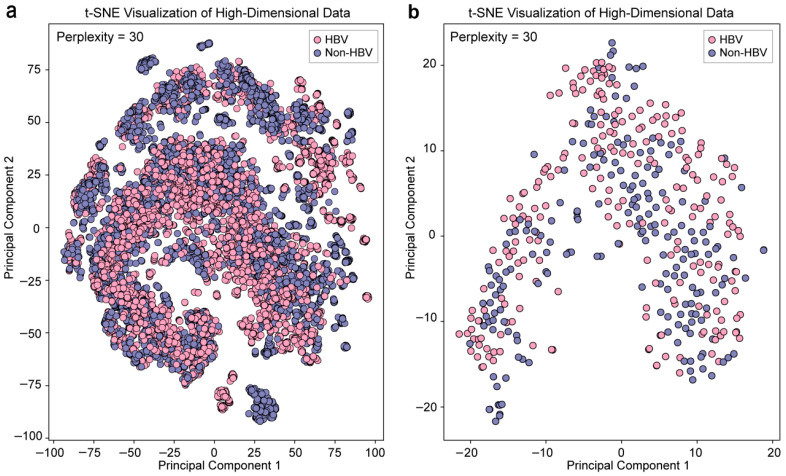
t-SNE analysis of serum protein profiles. Samples from HBsAg-positive HBV infection (pink) and HBsAg-negative (purple) groups are shown. (**a**) t-SNE plot generated from the full mass spectrometry spectrum. (**b**) t-SNE plot after data standardization and preprocessing.

Dimensionality reduction analysis was used to visualize the overall distribution patterns of serum fingerprints for HBsAg-positive and HBsAg-negative groups to assess the distinguish ability of the two sample types. Principal component analysis (PCA) and principal coordinate analysis (PCoA) were employed to reduce the dimensionality of the raw fingerprint data. According to the data, the HBsAg-positive and HBsAg-negative samples revealed a high degree of overlap and no apparent difference, indicating that the overall protein profiles of the two groups were very similar (Figure S1). A nonlinear technique, called t-SNE, was then used to improve pattern visualization in order to overcome the drawbacks of linear dimensionality reduction techniques. As shown in Figure 2a, although HBsAg-positive HBV infection samples (pink) and HBsAg-negative samples (purple) exhibit a slight separation trend, there is still significant overlap in the distribution areas of the two data sets, indicating that accurate differentiation is difficult to achieve using only the raw fingerprint information. This phenomenon remained evident when the dataset was further reduced for dimensionality reduction analysis. After dimensionality reduction, sample points from different groups exhibited an interlaced distribution, making it impossible to discern a stable discriminative pattern by visual inspection alone (Figure 2b). These results suggest that mass spectrometry fingerprints from samples with early or low-abundance HBV infection may possess only subtle and complex characteristic differences, making them difficult to discern directly by the naked eye.

### 3.3. Comparison of Preliminary Screening Models Based on Different Algorithms

**Figure 3:**
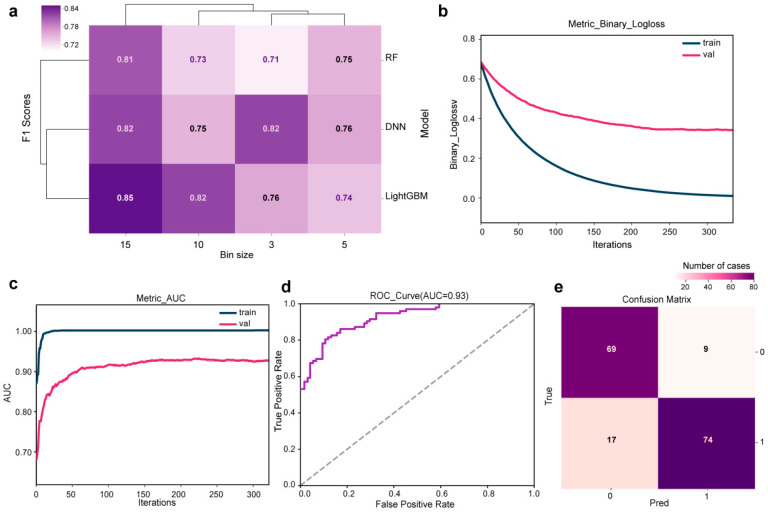
Development and evaluation of a preliminary screening model for HBsAg-positive HBV infection. (**a**) F1 scores of the model across different experimental conditions. (**b**) Binary logloss trajectory during model training. (**c**) AUC during training. (**d**) Final model AUC on the test set. (**e**) Confusion matrix of model predictions, where 0 denotes HBsAg-positive and 1 denotes HBsAg-negative.

As show in Figure 3a, three different algorithms—RF, DNN, and LightGBM—were employed to train models on datasets processed with various bin sizes. RF-based models achieved an F1 scores of 0.71, 0.75, and 0.73 on the subsets corresponding to bin size of 3, 5, and 10 *m*/*z*, respectively. Notably, performance improved significantly on the 15 *m*/*z* subset, yielding an F1 score of 0.81. Meanwhile, DNN-based models demonstrated more consistent performance, with F1 scores from 0.82 to 0.75 for the 3 to 15 *m*/*z* subsets. Overall, LightGBM-based models exhibited the highest diagnostic performance, achieving the F1 scores of 0.76, 0.74, 0.82, and 0.85 for bin size of 3, 5, 10, and 15 *m*/*z*, respectively. The corresponding AUC and confusion matrix are presented in Figures S2 and S3. Consequently, LightGBM was selected for subsequent analysis. The model incorporating the entire atlas was constructed using a bin size of 15 *m*/*z*. Based on this model, a preliminary prediction model for screening HBsAg-positive HBV infection was created.

During training, both the training loss and the validation loss decreased steadily within the first 150 iterations, indicating that the preliminary prediction model was effectively learning generalizable patterns from the data (Figure 3b). The model’s increasing capacity to discriminate between positive and negative samples was demonstrated by the AUC, which rose steadily and quickly until peaking at 0.93 (Figure 3c,d). At this performance peak (iteration 150), the model attained an F1 score of 0.85. However, beyond this point, the validation loss began to rise while the training loss continued to fall, signaling the onset of overfitting. We therefore employed early stopping in all subsequent training runs to automatically select the optimal model. Evaluation of this final model via a confusion matrix demonstrated a balanced performance on the test set, with a sensitivity of 89.2% and a specificity of 80.2% (Figure 3e).

### 3.4. Optimization of the Preliminary Prediction Models

To refine the preliminary prediction model, we optimized its performance by determining the minimum number of spectral replicates required for stable and reliable predictions. Technical variability inherent to MALDI-TOF MS measurements can introduce random noise that affects model stability. Identifying the point at which additional replicates no longer produced significant performance gains allowed a balance between spectral reliability and acquisition efficiency, reducing unnecessary data collection. As shown in Figure S4, the *predict* value increased steadily from 0.10 ± 0.03 with a single spectrum to 0.91 ± 0.04 with 24 replicates. The performance gain began to plateau at 16 replicates, with a *predict* value of 0.89 ± 0.06. Although the maximum accuracy reached 0.92 ± 0.03 with additional replicates, statistical analysis confirmed no significant improvement beyond 16 replicates (*p* = 0.56), indicating that further measurements provided negligible benefit. Based on these findings, 16 technical replicates per sample were adopted as the optimal standard for subsequent MALDI-TOF MS acquisitions. Building upon this optimized acquisition protocol, we proceeded to develop the screening model for HBsAg-positive HBV infection.

**Figure 4:**
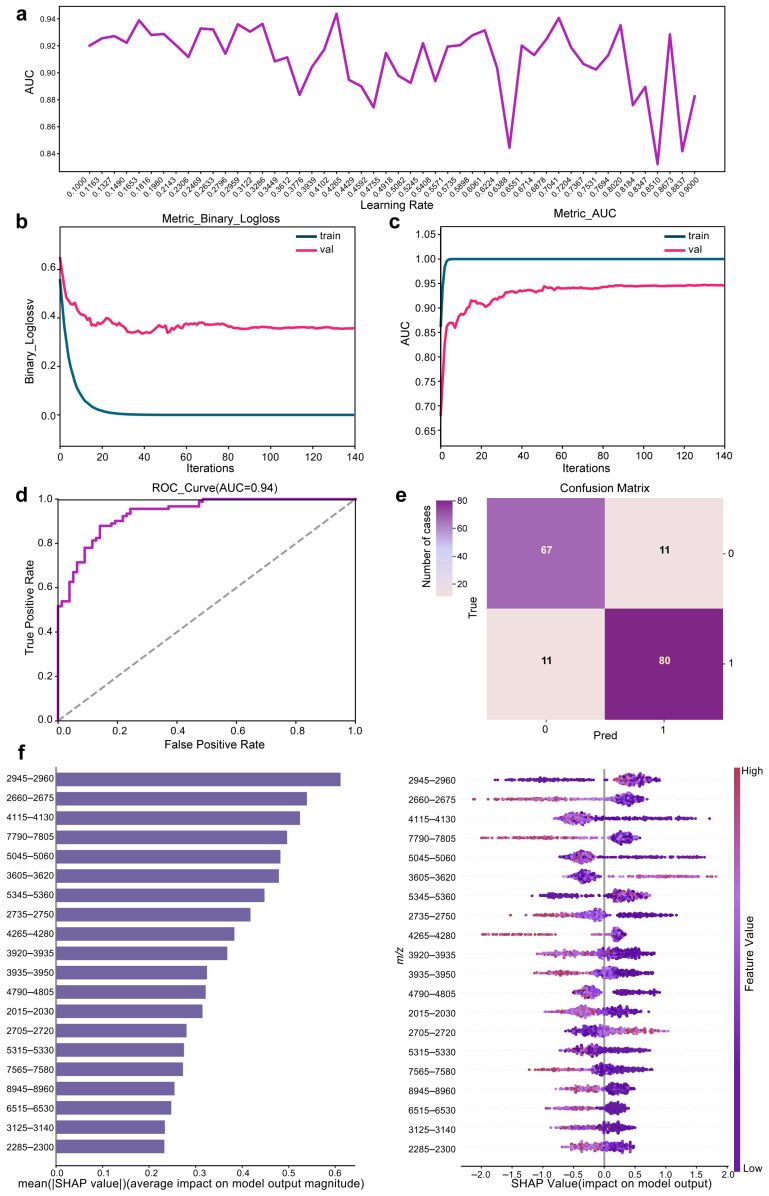
Optimization and evaluation of the screening model for HBsAg-positive HBV infection. (**a**) Screening performance across different learning rate. (**b**) Training trajectory measured by binary logloss. (**c**) AUC throughout the training process. (**d**) Final model performance assessed by the AUC. (**e**) Confusion matrix showing classification outcomes (0: HBsAg-negative; 1: HBsAg-positive). (**f**) Feature importance analysis based on SHAP value (red: high impact; purple: low impact).

The preliminary prediction model reached peak performance at iteration 150, achieving an AUC of 0.93 and an F1 score of 0.85. Early stopping was applied at this peak to terminate training, effectively preventing overfitting and establishing the model’s optimal baseline performance. Building upon this foundation and the optimized acquisition protocol, we next refined the preliminary prediction model by tuning the learning rate, a critical hyperparameter that directly influences model convergence and generalization. Proper adjustment of the learning rate is essential, as excessively small values may lead to slow convergence or entrapment in local optima, while overly large values can cause unstable training and poor generalization. As shown in Figure 4, systematic tuning across learning rates from 0.10 to 0.90 yielded substantial performance variation, with AUC ranging from 0.83 to 0.94. The optimal learning rate of 0.42 achieved peak performance, attaining an AUC of 0.94 (Figure 4a,d) and an F1 score of 0.87. Moreover, the model exhibited high stability in both logloss and AUC, achieving a consistent fit after the 39th iteration (Figure 4b,c). Evaluation via confusion matrix confirmed the model’s balanced predictive capability, with test-set sensitivity and specificity of 87.9% and 85.9%, respectively (Figure 4e).

To enhance interpretability and identify spectral features driving model decisions, we quantified the relative contributions of key characteristics using SHAP analysis. Figure 4f provides an overview of the features with the largest average contributions for each data point in the prediction model, along with their corresponding SHAP values. This analysis highlighted 20 key peak regions (2015–2030, 2285–2300, 2660–2675, 2705–2720, 2735–2750, 2945–2960, 3125–3140, 3605–3620, 3920–3935, 3935–3950, 4115–4130, 4265–4280, 4790–4805, 5045–5060, 5315–5330, 5345–5360, 6515–6530, 7565–7580, 7790–7805, 8945–8960 *m*/*z*) as the primary drivers of the model’s predictions. The optimized model demonstrated strong predictive performance, with well-calibrated sensitivity and specificity. Based on these results, it was designated as the HBsAg-positive HBV infection prediction model. However, features identified from a single SHAP analysis may include dataset-specific noise and may not generalize reliably. To improve robustness and ensure broader clinical applicability, the HBsAg-positive screening model was subsequently developed using a rigorous feature stabilization procedure.

### 3.5. Enhancement of the Generalization Capabilities

**Figure 5:**
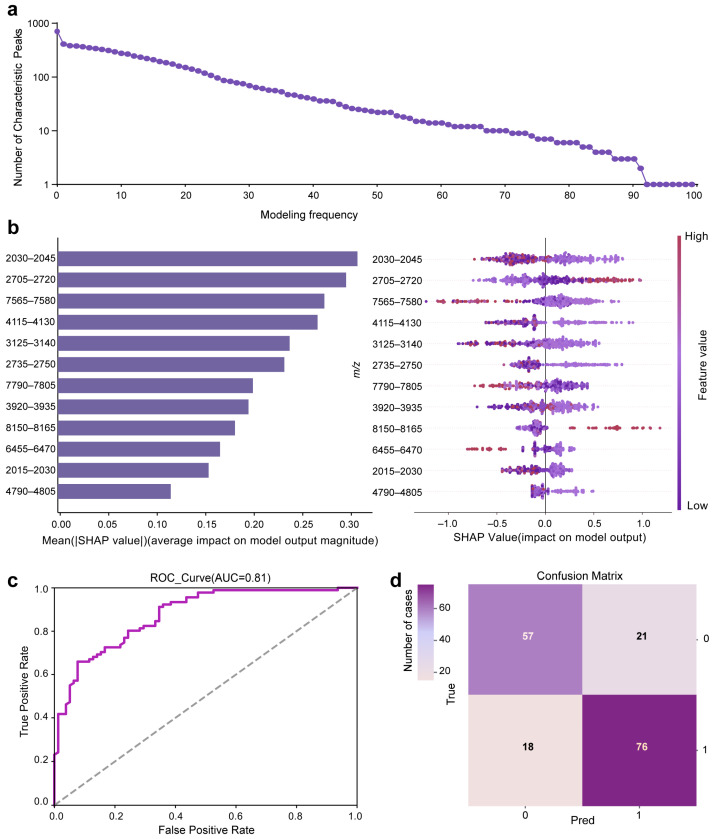
Generalization performance of the screening model for HBsAg-positive HBV infection. (**a**) Stability of feature selection across 100 bootstrap models, shown by the number of features retaining non-zero SHAP values. (**b**) SHAP summary plot of the top contributing features in the final model. (**c**) ROC curve showing the model’s discriminative ability. (**d**) Confusion matrix summarizing prediction outcomes on the test set (0: HBsAg-negative; 1: HBsAg-positive).

Building upon the learning rate-optimized model, we next addressed feature stability—a critical factor for clinical generalizability. While the optimized model demonstrated strong performance, its feature set derived from single-shot SHAP analysis may incorporate dataset-specific noise rather than truly generalizable biomarkers. To enhance robustness, we implemented a 100-iteration SHAP-based stability assessment, constructing independent models with distinct random seeds and calculating SHAP values for the top 20 features in each iteration. Features consistently contributing across bootstrap samples are more likely to represent authentic HBV-associated signals, while noise-driven features appear sporadically and are filtered out. As shown in Figure 5a, the number of features with non-zero SHAP contributions decreased exponentially with increasing model iterations, reaching a plateau after approximately 66 runs. The spectral regions that consistently contributed across these models were identified as stable fingerprints at 2015–2030, 2030–2045, 2705–2720, 2735–2750, 3125–3140, 3920–3935, 4115–4130, 4790–4805, 6455–6470, 7565–7580, 7790–8050 and 8150–8165 *m*/*z*, with mean contribution scores of 0.16, 0.31, 0.30, 0.23, 0.24, 0.20, 0.27, 0.12, 0.17, 0.27, 0.20 and 0.18, respectively (Figure 5b). These robust features were incorporated to build the final screening model for HBsAg-positive HBV infection, which exhibited enhanced predictive stability and interpretability.

The final model exhibited lower performance than the learning rate-optimized model, with the AUC decreasing from 0.94 to 0.88 and the F1 score from 0.87 to 0.79. This performance difference is attributable to the removal of unstable, dataset-specific features during consensus-based filtering, which reduced apparent training performance but improved model stability and generalizability. In independent validation, the final model achieved an AUC of 0.88 (Figure 5c), F1 score of 0.79, and a sensitivity and specificity of 77.7% and 76.0%, respectively (Figure 5d), providing a conservative estimate of expected clinical performance. To assess the robustness of the final model, five-fold cross-validation was performed, yielding a mean performance index of 0.79 ± 0.02. The low variability across folds indicates limited dependence on the specific composition of the training set. The cross-validated F1 score closely matched the independent validation result, further supporting the stability and generalizability of the final model.

### 3.6. Efficacy Evaluation of the Final Screening Model for HBsAg-Positive HBV Infection

The diagnostic performance of the final model was further evaluated in an independent cohort of 20 volunteers, all of whom also underwent conventional serological and molecular testing for HBV infection via ELISA and qPCR, alongside validation with our established final screening model (Table S2). ELISA identified 12 HBsAg-positive cases, yielding a 60.00% positive rate, with median marker levels of HBsAg at 2130.32 IU/mL, HBsAb at 0.64 IU/L, HBeAg at 0.39 S/CO, HBeAb at 0.02 S/CO, and HBcAb at 6.87 S/CO. Meanwhile, qPCR detected 7 HBV-positive individuals, with a median HBV DNA concentration of 2.65 × 10^3^ IU/mL. Consistent with the ELISA results, ML-MALDI-TOF MS correctly classified 12 positive samples. Using the ELISA (HBsAg) results as the reference, pairwise comparisons (*p* < 0.05) showed that qPCR exhibited a sensitivity of 58.3% (7/12; 95% CI: 27.7–84.8%) and a specificity of 100.0% (8/8; 95% CI: 63.1–100.0%), whereas our ML-MALDI-TOF MS demonstrated significantly higher sensitivity of 91.7% (11/12; 95% CI: 61.5–99.8%) with a specificity of 75.0% (6/8; 95% CI: 34.9–96.8%). ML-MALDI-TOF MS detected four additional HBsAg-positive cases that were missed by qPCR (Tests 2, 6, 8, and 10), while producing one false-negative (Test 12) and two false-positive results (Tests 13 and 14) (Table 1).

No statistically significant difference was observed in the overall screening performance among ML-MALDI-TOF MS, qPCR and ELISA (*p* = 0.06). Pairwise comparisons using McNemar’s test with continuity correction (*p* < 0.05) revealed no significant difference between qPCR and ELISA (*p* = 0.07) or between ML-MALDI-TOF MS and ELISA (*p* = 0.32). Notably, a significant difference was detected between ML-MALDI-TOF MS and qPCR (*p* = 0.046), demonstrating the superior sensitivity of ML-MALDI-TOF MS compared to qPCR. The non-overlapping 95% confidence intervals for sensitivity further substantiate the inherent clinical advantage of ML-MALDI-TOF MS in detecting HBsAg-positive HBV infection.

To further validate the specificity of our HBsAg-positive HBV screening model, we performed control experiments using samples from patients with syphilis or HCV (Test 21, Test 22), purified HBV viral particles (Test 23–Test 25), and purified antigens and antibodies of HBV (Test 26–Test 30). As shown in Table 2, all control samples were correctly identified as negative. The HBV viral particles did not produce any spectral peaks, while the purified antigens/antibodies and the syphilis/HCV samples generated peaks that did not align with the model’s specific feature set. These results indicate that the algorithm yielded no positive results for isolated molecular targets, including purified HBV viral particles, recombinant HBsAg, HBeAg, HBcAg, or serum samples from patients with syphilis or HCV infection, confirming that the assay specifically detects HBsAg-positive HBV infection rather than non-specific viral or antigenic components.

## 4. Discussion

HBV infection is a major global health concern, as it significantly increases the risk of cirrhosis and hepatocellular carcinoma, contributing to high morbidity and mortality rates. Timely and accurate diagnosis is therefore critical for effective disease management and public health control. In clinical settings, ELISA and qPCR remain the most widely adopted techniques for HBV detection [20,21]. Despite their established role, widespread adoption faces several obstacles, including the high expense of testing, the strict criteria for shipping and storage, and the need for operators with specialized skills [22].

MALDI-TOF MS has emerged as a promising alternative that addresses many of these limitations [23]. We compared its performance of this optimized ML-MALDI-TOF MS workflow with conventional qPCR and ELISA (Table 1). The results show several key advantages of ML-MALDI-TOF MS: a rapid detection time of approximately 1 min; a low cost of ~$0.14 per sample; minimal reagent consumption, requiring only a standard solution and 70% formic acid, and relaxed storage conditions allowing for room-temperature preservation. Crucially, the simplified serum fingerprint acquisition workflow eliminates complex pretreatment steps such as protein extraction or amplification, enabling direct sample analysis with minimal manipulation. This streamlined approach reduces technical variability, shortens hands-on time, and allows a single operator to oversee the entire screening chain from sample preparation and data acquisition to result interpretation, thereby enhancing traceability, reproducibility, and quality control in clinical screening settings.

In the development of ML-based disease screening methods, various algorithms have been developed, including partial least squares discriminant analysis, logistic regression, support vector machine, RF, and Naive Bayes [24,25]. Given the distinct theoretical foundations and learning mechanisms of these models, their diagnostic performance can vary substantially; thus, direct comparative evaluation is essential to identify the optimal approach [26]. In the present study, three algorithms were selected based on their complementary suitability for the low-sample characteristics of the dataset (~253 training samples; feature-to-sample ratios of 4.7 at binsize = 15 and 23.7 at binsize = 3): LightGBM, whose histogram-based discretisation efficiently processes ~1200 to ~6000 spectral features and whose regularisation parameters directly constrain model complexity relative to the limited training set. RF, whose bootstrap aggregation and random feature subsampling prevent overfitting to noise-dominated *m*/*z* channels when features substantially outnumber samples. And DNN, whose multilayer nonlinear architecture captures cross-channel composite features arising from the physical continuity of MALDI-TOF MS, wherein the same protein produces correlated intensity patterns across multiple adjacent *m*/*z* channels.

The comparative analysis demonstrated that LightGBM consistently outperformed RF and DNN (Figure 3 and Figures S2 and S3). This performance advantage derives from LightGBM’s gradient boosting framework, which differs fundamentally from RF and DNN architectures. Unlike RF, which constructs independent decision trees and aggregates their predictions through averaging or voting [27], LightGBM constructs decision trees sequentially, allowing each new tree to correct the errors of its predecessors and thereby refine decision boundaries for subtle spectral patterns associated with HBV infection. Compared with DNNs, which integrate all input features and are more susceptible to irrelevant or noisy variables, LightGBM performs implicit feature selection during tree construction, retaining only informative *m*/*z* features. This characteristic is particularly advantageous for high-dimensional MALDI-TOF MS data, where most spectral channels represent background noise. Moreover, its leaf-wise growth strategy and histogram-based discretization enable more effective modeling of non-linear feature interactions and improve robustness to minor peak shifts and technical variability [28,29]. Consequently, LightGBM provides an effective balance between predictive accuracy, computational efficiency, and robustness for MALDI-TOF spectral classification in low-sample settings.

Several measures were implemented to mitigate overfitting risk given the high-dimensional, low-sample nature of the dataset. At the data level, all 24 technical replicate spectra from each participant were averaged prior to model input, stabilising the input feature distribution and reducing within-sample variance that could otherwise introduce noise-driven patterns during training. Dataset partitioning was performed strictly at the patient level, ensuring that all spectra from a given participant were assigned exclusively to either the training or test set, thereby preventing any form of data leakage between partitions. At the training level, five-fold cross-validation was applied across all three models to provide robust estimates of generalisation performance and reduce dependence on any single data split. Additionally, early stopping was configured to terminate training after 100 consecutive epochs without improvement in binary logloss on the validation fold, preventing continued weight updates beyond the point of generalisable learning. To further ensure the stability of feature importance rankings—which are sensitive to stochastic elements such as weight initialisation and data shuffling—each model was trained across multiple random seeds, and only features demonstrating consistent contributions across runs were retained for downstream biomarker analysis. Collectively, these measures address overfitting at the data, partitioning, training, and feature selection levels, supporting the reliability of the reported classification performance and biomarker candidates despite the moderate sample size.

Consistent with previous serological studies, the peak intensity of serum-based assays was lower than that of traditional microbiological assays. We observed fluctuations in peak intensities during spectral acquisition, potentially due to instrumental variability [30]. To address these challenges, we introduced—for the first time in serum marker detection—a data management strategy based on variable bin sizes (Figure 3), a method originally developed for microbiological detection [11]. This approach proved effective in mitigating errors caused by peak shifts and improved the overall stability of spectral profiles. To further minimize variability between samples, several data preprocessing strategies were explored. Initial outlier removal proved ineffective due to region-specific spectral variations. To address this, we developed a single-sample aggregation and standardization protocol that integrates replicate spectra by summation, averaging, or maximization. This approach improved discrimination between HBsAg-positive and HBsAg-negative samples and reduced errors from sample heterogeneity (Figure 2). Single-sample aggregation and standardization provide a novel preprocessing strategy for MALDI-TOF MS data, effectively mitigating variability in serum proteomic analysis.

The preliminary prediction model exhibited instability in its early iterations, suggesting an over-reliance on features that lacked generalizability. To address this, we implemented a novel strategy that built 100 independent models using distinct random seeds and retained only HBV-associated features with consistently non-zero SHAP values across most iterations (Figure 5a). In this framework, noise-driven features showed highly variable and randomly distributed SHAP contributions, whereas biologically relevant features maintained stable importance patterns, enabling effective elimination of spurious signals. Ensemble averaging further reduced the influence of random initialization, resulting in a final model with a unified and reproducible screening logic independent of stochastic effects. As a result, this strategy improves robustness to operational deviations and instrument variability while preserving sensitivity to subtle spectral differences associated with early-stage HBV infection. This ensemble method effectively mitigated overfitting and enhanced model robustness. From this process, we identified a panel of serum peptide biomarkers strongly associated with HBsAg-positive HBV infection (Figure 5b and Figure S5). This refinement resulted in a moderate reduction in model performance compared with the preliminary model, with the AUC decreasing from 0.94 to 0.88 and the F1 score from 0.87 to 0.79. However, the refined model showed improved feature stability and generalizability. The observed performance difference is attributable to the exclusion of unstable, dataset-dependent features that contributed to higher apparent accuracy in the preliminary model but did not generalize across model iterations. As a result, the performance estimates of the refined model provide a more realistic assessment of expected performance in independent datasets. This refined approach yields a more reliable tool for HBsAg-positive screening, highlighting the value of ensemble strategies in improving the generalizability of clinical prediction models.

Clinical specificity validation experiments revealed that neither purified HBV viral particles (Tests 23–25) nor purified HBsAg (Test 30) were classified as positive by the proposed model (Table 2). Although the model was trained using HBsAg-positive serum samples, these results indicate that the classification is not driven by direct detection of viral components. The MALDI-TOF MS-based serum fingerprinting approach applied in this study captures host-derived proteomic alterations associated with HBV infection rather than viral antigens themselves. These alterations include infection-induced changes in acute-phase proteins, immune-related peptides, and complement fragments, which collectively generate characteristic spectral patterns within the 2–20 kDa detection range. In contrast, purified viral particles lack the host serum context required to produce these infection-associated proteomic signatures and therefore do not generate the spectral features learned by the model. Similarly, purified HBsAg, with a molecular weight of approximately 24–27 kDa, lies outside the effective detection window and does not contribute detectable signal peaks. Consistent with this interpretation, the model’s discriminatory features, including the stable signals observed in the 2015–2030 Da region (Figure 4f and Figure 5b), are attributable to host-derived peptides rather than viral proteins. Collectively, these results demonstrate that the proposed method identifies the physiological state of HBV infection as reflected by host response patterns, rather than directly detecting viral material. Notably, the absence of false-positive classifications for purified viral components further supports the assay’s specificity and reduces the likelihood of erroneous detection in scenarios involving acellular viral material, such as vaccination-related antigen exposure or environmental contamination.

The integration of MALDI-TOF MS and ML enables the discovery of biomarkers and demonstrates promising performance for large-scale HBV screening. Despite these advances, several challenges remain. The limited representativeness of our database, attributable to the restricted sample size, narrow source range, and focus on a single disease, hinders the comprehensive characterization of HBV infection heterogeneity. Expanding the sample pool to encompass larger and more diverse cohorts is essential for improving the model’s diagnostic accuracy and generalizability. Furthermore, the molecular identities of the distinctive spectral peaks remain uncharacterized, and diagnostic accuracy for individual-level confirmation remains suboptimal, underscoring the need for further methodological refinement. Future studies should employ tandem mass spectrometry and integrate complementary spectroscopic techniques, including Fourier transform infrared spectroscopy, Raman spectroscopy, surface-enhanced laser desorption/ionization MS, and near-infrared spectroscopy, to elucidate the composition of these peaks. Such approaches would provide deeper insights into both molecular profiles, such as peptide and protein signatures, and global biochemical characteristics, including lipid, carbohydrate, and protein secondary structures. This would facilitate the identification of specific biomarkers and support the advancement of next-generation HBV screening and diagnostics. As the methodology matures, the ML-MALDI-TOF MS platform could be extended to detect other pathogens and elusive biomarkers, thereby broadening its clinical utility and enhancing diagnostic reliability, including the ability to distinguish infection stages or detect occult infections. Such developments hold significant potential to open new avenues in disease control and enhance public health security.

## 5. Conclusions

This study establishes MALDI-TOF MS combined with optimized ML algorithms as a rapid, cost-effective, and high-throughput tool for population-level screening of HBsAg-positive HBV infection. Through comparative analysis of multiple algorithms, LightGBM was identified as the optimal classifier, further enhanced by variable bin size processing, single-sample aggregation, and standardization. A novel 100-iteration randomization strategy was developed to identify stable fingerprint features. In the independent validation cohort, the final model achieved an AUC of 0.88, an F1 score of 0.79, and sensitivity and specificity of 77.7% and 76.0%, respectively. Importantly, this method offers substantial practical advantages over conventional assays. With a detection time of approximately 1 min per sample and a per-sample cost of ~$0.14, combined with the absence of reliance on precise temperature-controlled equipment, this approach represents a highly feasible technical solution for rapid HBsAg-positive population screening in resource-limited settings. These findings position ML-MALDI-TOF MS as a novel supplementary tool for HBV public health prevention and control, while also establishing a foundation for broader application of this platform in large-scale screening for other infectious diseases.

## Figures and Tables

**Table 1:** Results of different methods for screening HBV infection.

	Samples	qPCR	HBsAg-ELISA	ML-MALDI-TOF MS
Methods				
Test 1		Positive	Positive	Positive
Test 2		Negative	Positive	Positive
Test 3		Positive	Positive	Positive
Test 4		Positive	Positive	Positive
Test 5		Positive	Positive	Positive
Test 6		Negative	Positive	Positive
Test 7		Positive	Positive	Positive
Test 8		Negative	Positive	Positive
Test 9		Positive	Positive	Positive
Test 10		Negative	Positive	Positive
Test 11		Negative	Positive	Positive
Test 12		Positive	Positive	Negative
Test 13		Negative	Negative	Positive
Test 14		Negative	Negative	Positive
Test 15		Negative	Negative	Negative
Test 16		Negative	Negative	Negative
Test 17		Negative	Negative	Negative
Test 18		Negative	Negative	Negative
Test 19		Negative	Negative	Negative
Test 20		Negative	Negative	Negative

**Table 2:** Accuracy verification of the ML-MALDI-TOF MS established in this study.

Samples	Targets	Results
Test 21	Syphilis	Negative
Test 22	HCV	Negative
Test 23	HBV particles	Negative
Test 24		Negative
Test 25		Negative
Test 26	HBsAb	Negative
Test 27	HBcAb	Negative
Test 28	HBeAb	Negative
Test 29	HBeAg	Negative
Test 30	HBsAg	Negative

## Data Availability

The original contributions presented in this study are included in the article and Supplementary Material. Further inquiries can be directed to the corresponding authors.

## Supplementary Materials

The following supporting information can be downloaded at: https://www.mdpi.com/article/10.3390/microorganisms14030702/s1, Table S1: Baseline demographic characteristics of the study and control groups; Table S2: HBV infection screening results using various identification schemes; Figure S1: Multivariate analysis of serum profiles from HBsAg-positive and HBsAg-negative groups; Figure S2: Model performance across bin sizes and machine learning algorithms; Figure S3: Classification performance across bin sizes and machine learning algorithms; Figure S4: Correlation between the frequency of spectruming and *predict*; Figure S5: Distribution of key discriminatory features in the HBsAg-positive HBV infection screening model.
