# Supplementary material for: Machine Learning-Enhanced MALDI-TOF Mass Spectrometry for Screening HBsAg-Positive Patients
Source: Microorganisms. 2026 Mar 20;14(3):702. doi: 10.3390/microorganisms14030702 (PMC13028653; doi:10.3390/microorganisms14030702)
Supplement: Supplementary file 1 [file microorganisms-14-00702-s001.zip › microorganisms-4151129-supplementary.pdf]

Supplementary Materials

Machine Learning-Enhanced MALDI-TOF Mass Spectrometry for Screening HBsAg-Positive Patients

Tiantian Zhang [1,2,3,†], Shixuan Huang [2,3,†], Junxun Li [4,†], Yuwei Wu [2,3], Xinyu Zhao [2,3], He Gao [2,3], Juan Yang [2,3], Lingshuang Yang [2,3], Lulu Cao [2,3], Xinqiang Xie [2,3], Hui Zhao [2,3], Jing Cheng [4], Hongxia Tan [4], Ying Li [2,3,*] and Qingping Wu [1,2,3,*]

[1] School of Food and Biological Engineering, Shaanxi University of Science and Technology, Xi'an 710021, China; ttsghww@163.com

[2] State Key Laboratory of Applied Microbiology Southern China, Guangdong Provincial Key Laboratory of Microbial Safety and Health, National Health Commission Science and Technology Innovation Platform for Nutrition and Safety of Microbial Food, Key Laboratory of Big Data Technologies for Food Microbiological Safety, State Administration for Market Regulation, Institute of Microbiology, Guangdong Academy of Sciences, Guangzhou 510070, China; nhuhuganaxis@outlook.com (S.H.); 13503036950@163.com (Y.W.); justinzhaoxy9897@163.com (X.Z.); gaohe.881128@163.com (H.G.); juanayangon@163.com (J.Y.); yangls8272@163.com (L.Y.); 17754113380@163.com (L.C.); woshixinqiang@126.com (X.X.); zhaohuichinese@163.com (H.Z.)

[3] Food and Drug Laboratory, Guangdong Detection Center of Microbiology, Guangzhou 510070, China

[4] Department of Medical Laboratory, First Affiliated Hospital, Sun Yat-Sen University, Guangzhou 510080, China; lijunx5@mail.sysu.edu.cn (J.L.); chengj888@mail.sysu.edu.cn (J.C.); 13926439681@139.com (H.T.)

* Correspondence: liying@gdim.cn (Y.L.); wuqp203@163.com (Q.W.)

† These authors contributed equally to this work.

Supplementary Tables

Table S1. Baseline demographic characteristics of the study and control groups

Variables	HBsAg-positive group (N = 226)	HBsAg-negative group (N = 196)	*P* value
Age (years, mean ± SD)	51.23 ± 14.12	50.14 ± 16.80	0.40
Sex (male/female)	141/85	120/76	0.81
ALT (U/l), M (QR)	14.80	13.00	0.07
AST (U/l), M (QR)	21.00	17.85	0.06
ALB (g/l), M (QR)	43.30	41.90	0.28
DBIL (μmol/l), M (QR)	4.20	4.23	0.05
TBIL (μmol/l), M (QR)	13.00	11.60	0.16

Note: The data is shown as mean ± standard deviation (mean ± SD).

Abbreviations: M (QR), median (interquartile interval); ALT, alanine aminotransferase; AST, aspartate aminotransferase; ALB, albumin; DBIL, direct bilirubin; TBIL, total bilirubin.

Table S2. HBV infection screening results using various identification schemes

Samples	qPCR	ELISA				
	HBV DNA (IU/mL)	HBsAg (IU/mL)	HBsAb (IU/L)	HBeAg (S/CO)	HBeAb (S/CO)	HBcAb (S/CO)
Test 1	1.14×10^7	2152.32	0.01	0.3	0.02	7.52
Test 2	< 100	10.56	35.24	0.39	0.02	6.87
Test 3	5.11×10^3	11499.62	0.64	0.39	0.03	6.87
Test 4	2.65×10^3	48468.04	0	735.23	39.1	5.55
Test 5	2.45×10^2	42.59	0	0.42	0.02	6.65
Test 6	< 100	0.2	1.24	0.3	0.02	6.86
Test 7	3.88×10^8	38573.41	0.06	0.43	0.02	7.37
Test 8	< 100	2108.31	1.55	0.42	0.05	6.47
Test 9	1.82×10^9	111309.14	0.64	1417.33	36.63	5.93
Test 10	< 100	2049.29	0.77	0.32	0.02	7.53
Test 11	< 100	228.96	0.66	0.3	0.01	7.25
Test 12	7.01×10^4	2616.46	0.19	0.31	0.01	7.37
Test 13	-	-	36.96	-	45.14	-
Test 14	-	-	30.63	-	35.15	-
Test 15	-	-	74.28	-	63.26	-
Test 16	-	-	64.21	-	51.14	-
Test 17	-	-	52.71	-	42.13	-
Test 18	-	-	55.53	-	44.1	-
Test 19	-	-	36.26	-	36.63	-
Test 20	-	-	66.29	-	28.36	-

Supplementary Figure

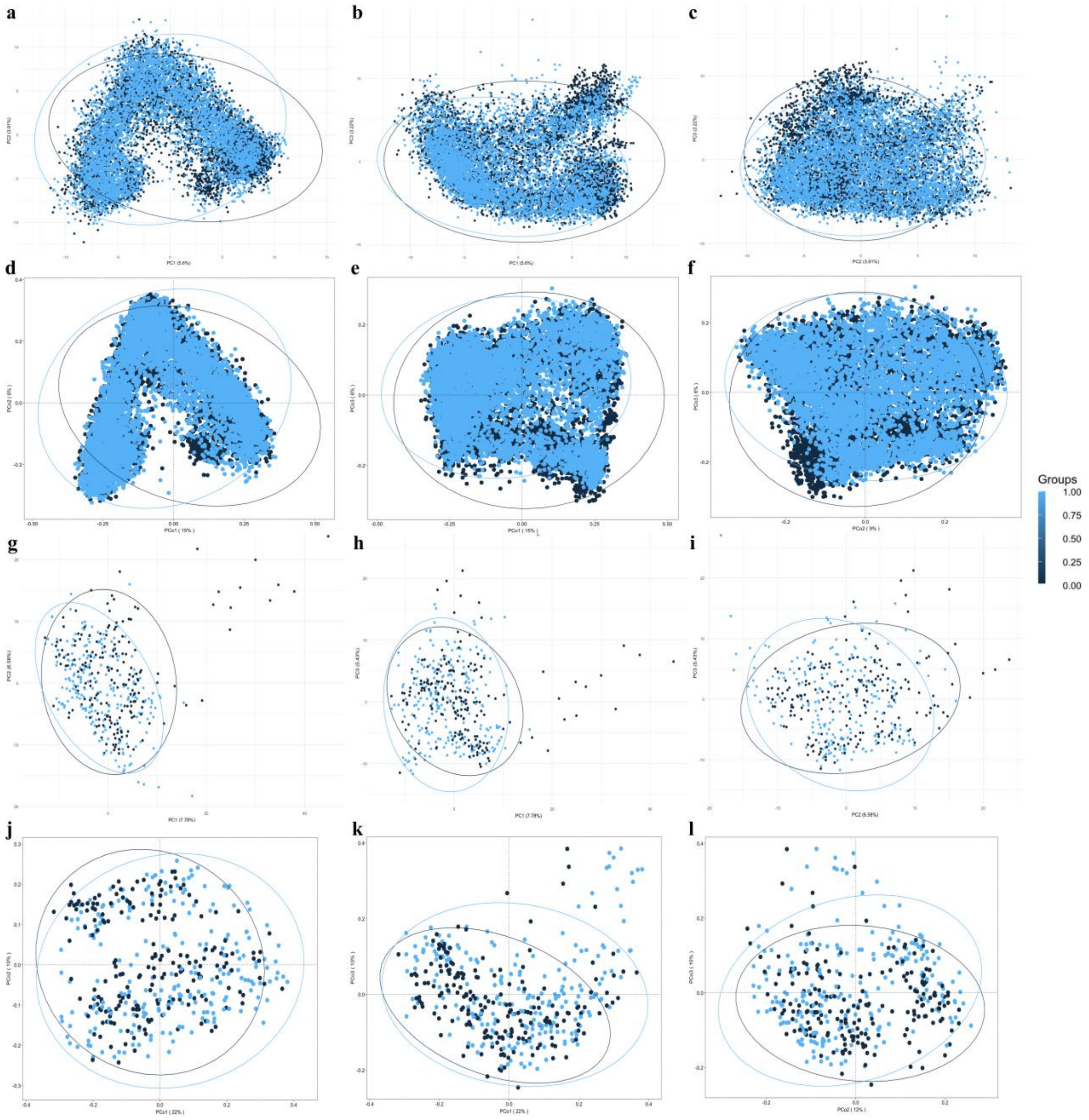

Figure S1. Multivariate analysis of serum profiles from HBsAg-positive and HBsAg-negative groups. Principal component analysis (PCA) and principal coordinate analysis (PCoA) were applied to compare the global fingerprint patterns between the HBsAg-positive (blue) and HBsAg-negative (black) groups. (a-c) PCA score plots of the raw data for component pairs: a, PC1 vs. PC2; b, PC1 vs. PC3; c, PC2 vs. PC3. (d-f) PCoA score plots of the raw data for coordinate pairs: d, PCo1 vs. PCo2; e, PCo1 vs. PCo3; f, PCo2 vs. PCo3. (g-i) PCA score plots after

standardization and single-sample consolidation preprocessing for component pairs: g, PC1 vs. PC2; h, PC1 vs. PC3; i, PC2 vs. PC3. (j-l) PCoA score plots after standardization and single-sample consolidation preprocessing for coordinate pairs: j, PCo1 vs. PCo2; k, PCo1 vs. PCo3; l, PCo2 vs. PCo3.

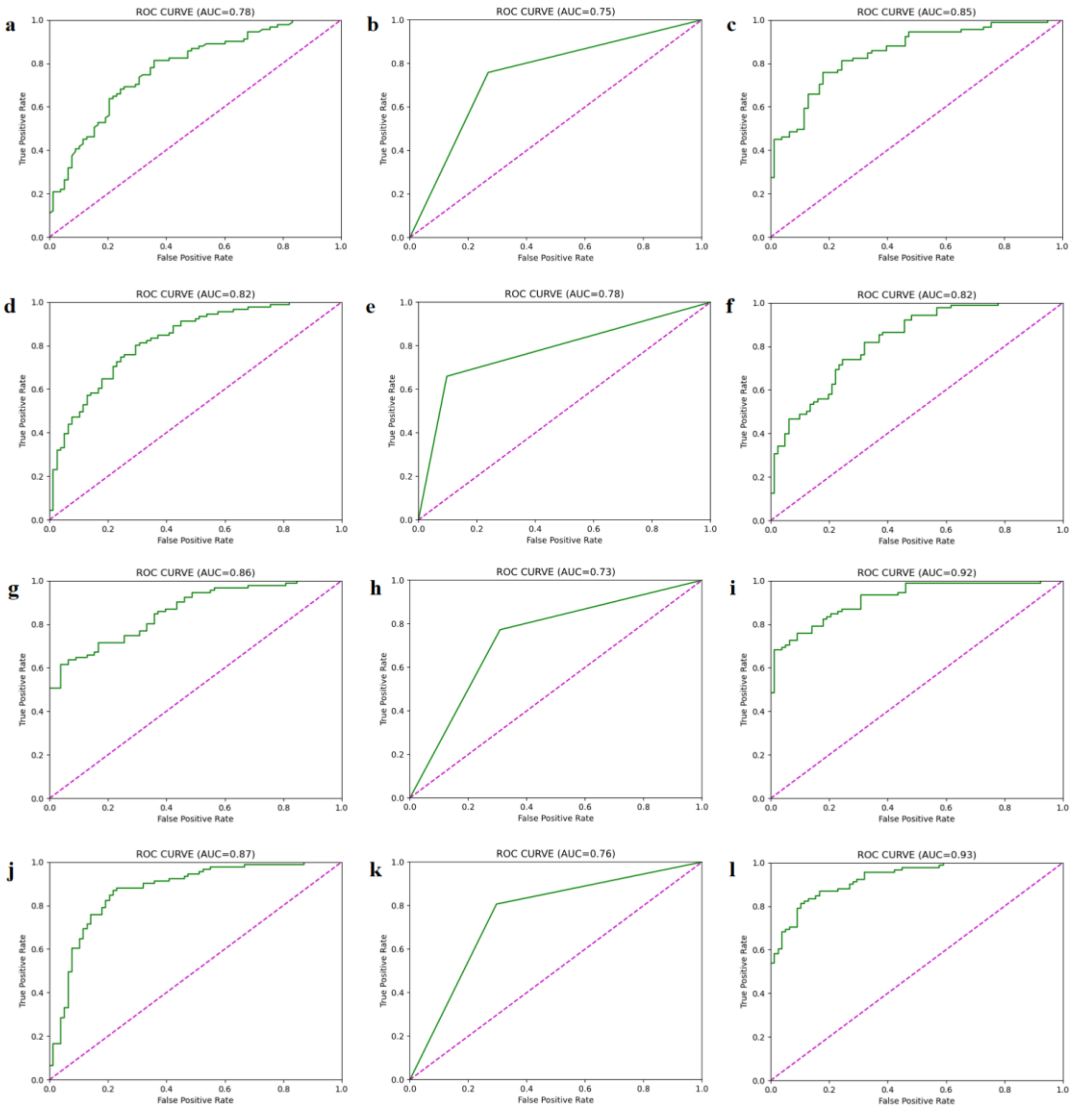

Figure S2. Model performance across bin sizes and machine learning algorithms. The area under the curve (AUC) was evaluated for three algorithms - Random Forest (RF), Deep Neural Network (DNN), and light gradient boosting machine (LightGBM) - using mass spectrometry data binned at four different bin sizes (3, 5, 10, and 15 *m/z*). (a-c) Performance at a bin size of 3: a, RF; b, DNN; c, LightGBM. (d-f) Performance at a bin size of 5: d, RF; e, DNN; f, LightGBM. (g-i) Performance at a bin size of 10: g, RF; h, DNN; i, LightGBM. (j-l) Performance at a bin size of 15: j, RF; k, DNN; l, LightGBM.

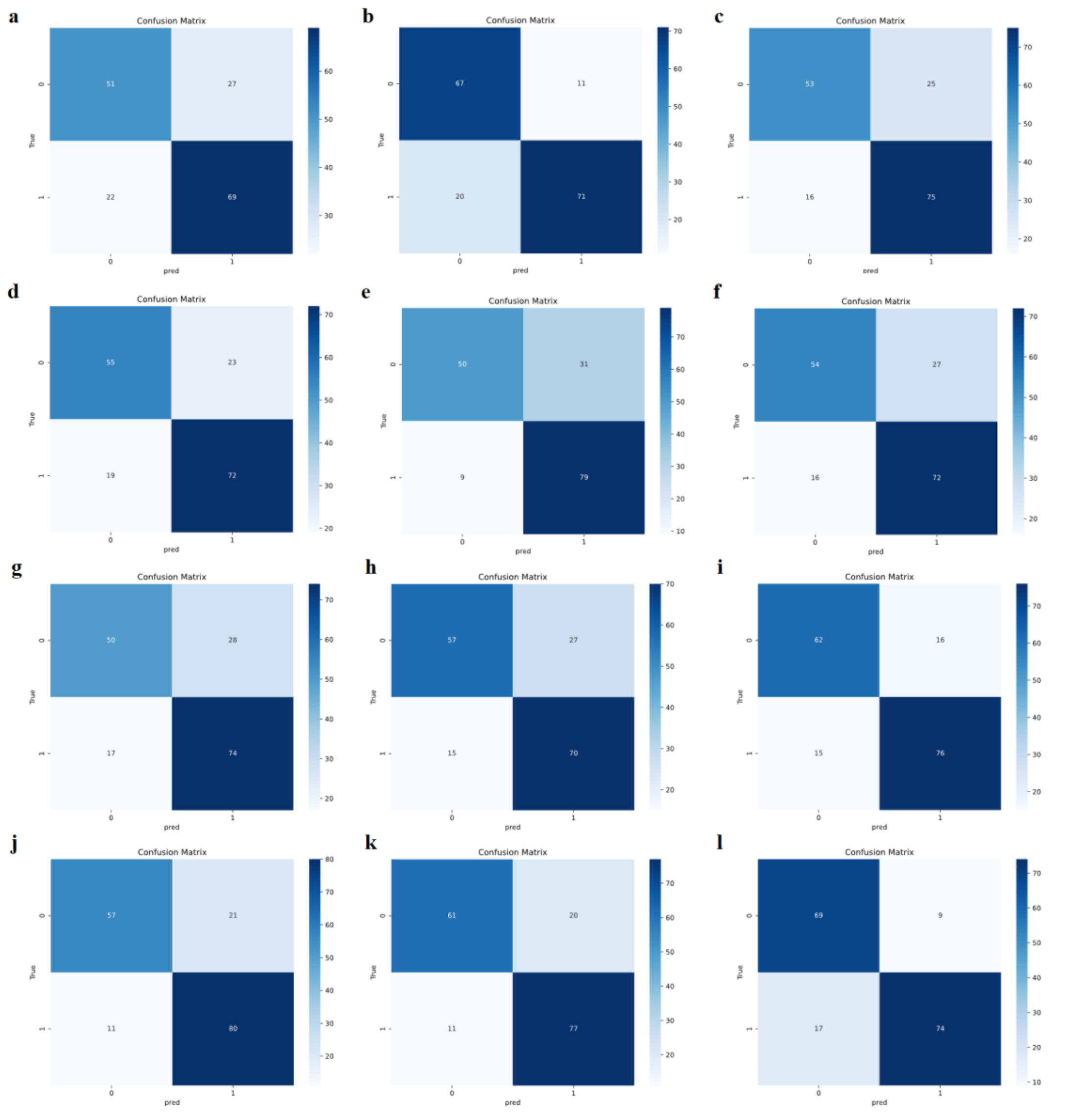

Figure S3. Classification performance across bin sizes and machine learning algorithms. Confusion matrices are shown for three algorithms - Random Forest (RF), Deep Neural Network (DNN), and light gradient boosting machine (LightGBM) - applied to mass spectrometry data processed with four different bin sizes. (a-c) Bin size = 3: a, RF; b, DNN; c, LightGBM. (d-f) Bin size = 5: d, RF; e, DNN; f, LightGBM. (g-i) Bin size = 10: g, RF; h, DNN; i, LightGBM. (j-l) Bin size = 15: j, RF; k, DNN; l, LightGBM.

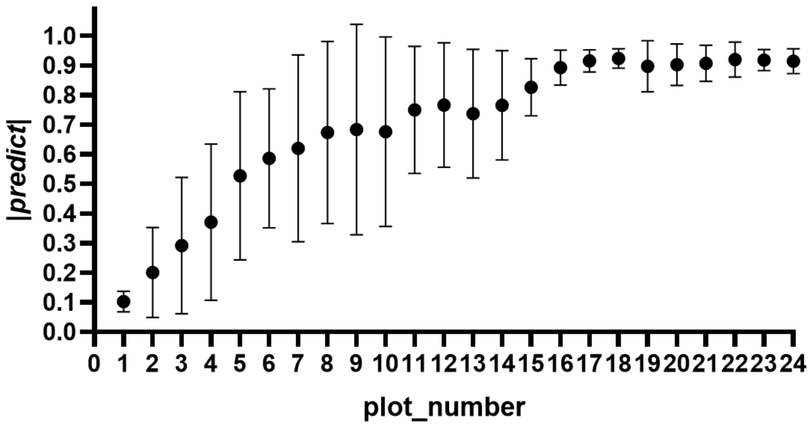

Figure S4. Correlation between the frequency of spectruming and *predict*.

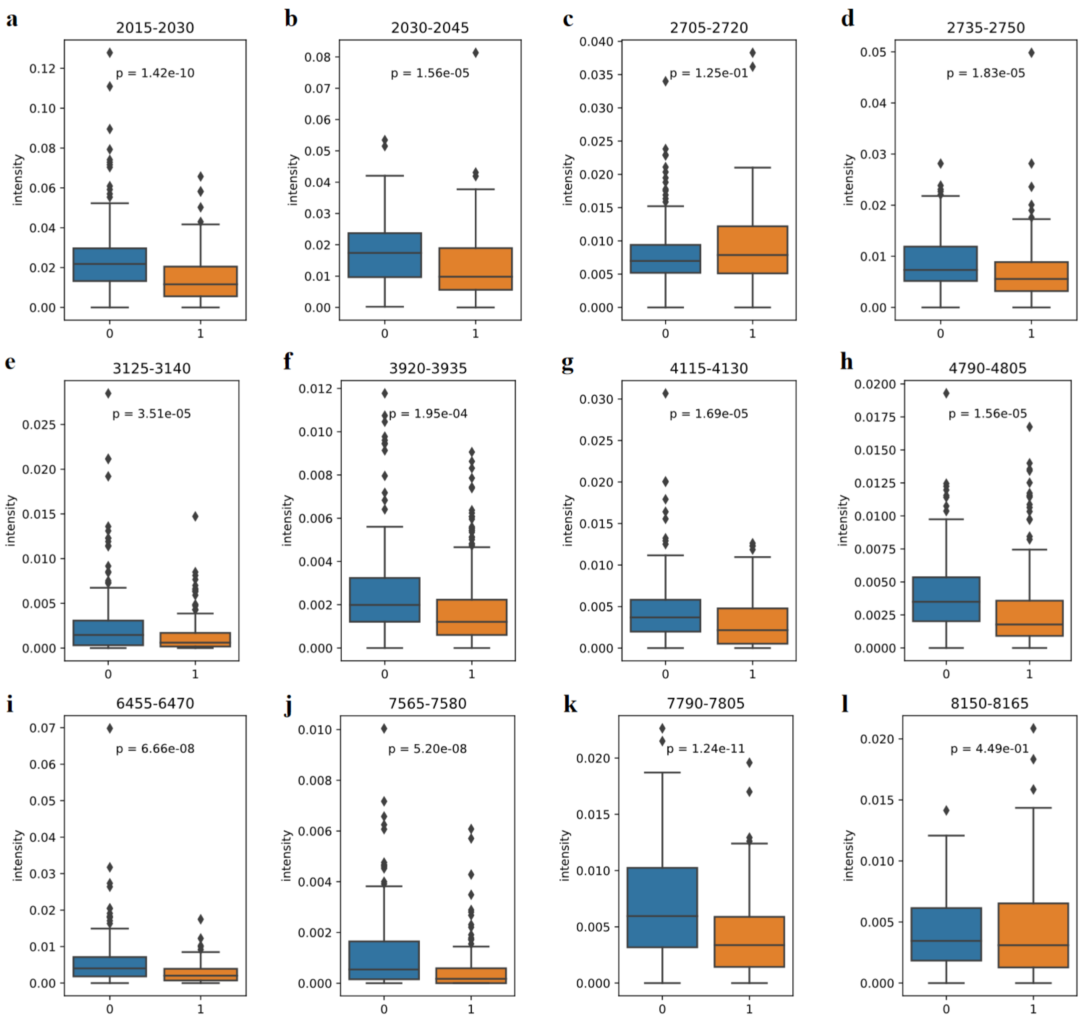

Figure S5. Distribution of key discriminatory features in the HBsAg-positive HBV infection screening model. Box plots show the ion intensity distributions for the twelve m/z features identified as most important for distinguishing HBsAg-positive from HBsAg-negative samples. (a) 2,015 - 2,030 *m/z*. (b) 2,030 - 2,045 *m/z*. (c) 2,705 - 2,720 *m/z*. (d) 2,735 - 2,750 *m/z*. (e) 3,125 - 3,140 *m/z*. (f) 3,920 - 3,935 *m/z*. (g) 4,115 - 4,130 *m/z*. (h) 4,790 - 4,805 *m/z*. (i) 6,455 - 6,470 *m/z*. (j) 7,565 - 7,580 *m/z*. (k) 7,790 - 8,050 *m/z*. (l) 8,150 - 8,165 *m/z*.
